# Prognostic effects of 25-hydroxyvitamin D levels in gastric cancer

**DOI:** 10.1186/1479-5876-10-16

**Published:** 2012-01-27

**Authors:** Chao Ren, Miao-zhen Qiu, De-shen Wang, Hui-yan Luo, Dong-sheng Zhang, Zhi-qiang Wang, Feng-hua Wang, Yu-hong Li, Zhi-wei Zhou, Rui-hua Xu

**Affiliations:** 1 State Key Laboratory of Oncology in South China, Guangzhou 510060, China; 2 Department of Medical Oncology, Sun Yat-sen University Cancer Center, 651 Dong Feng Road East, Guangzhou 510060, China; 3 Department of gastric and pancreatic surgery oncology, Sun Yat-sen University Cancer Center, Guangzhou 510060, China

**Keywords:** vitamin D, Gastric cancer, Prognosis, Elisa

## Abstract

**Background:**

Results from large epidemiologic studies on the association between vitamin D and gastric cancer are controversial. Vitamin D significantly promotes apoptosis in the undifferentiated gastric cancer cell, but the prognostic effects of its levels are unknown.

**Methods:**

197 gastric carcinoma patients who received treatment in the cancer centre of Sun Yat-sen University from January 2002 to January 2006 were involved in the study. The stored blood drawn before any treatment was assayed for 25-hydroxyvitamin D levels. The clinicopathologic data were collected to examine the prognostic effects of vitamin D.

**Results:**

The mean vitamin D levels of the 197 gastric patients was 49.85 ± 23.68 nmol/L, among whom 114(57.9%) were deficient in Vitamin D(< 50 nmol/L), 67(34%) were insufficient (50-75 nmol/L) and 16(8.1%) were sufficient (> 75 nmol/L). Clinical stage (*P *= 0.004) and lymph node metastasis classification (*P *= 0.009) were inversely associated with vitamin D levels. The patients with high vitamin D levels group (≥ 50 nmol/L) had a higher overall survival compared with the low vitamin D levels group (< 50 nmol/L)(*P *= 0.018). Multivariate analysis indicated that vitamin D levels were an independent prognostic factor of gastric cancer (*P *= 0.019).

**Conclusions:**

Vitamin D deficiency may be associated with poor prognosis in gastric cancer.

## Background

Gastric cancer is the fourth most common cancer and the second leading cause of cancer-related deaths following lung carcinoma despite a worldwide decline in both incidence and mortality since the later half of the twentieth century [1]. Although in most Western countries the incidence lies between 10 and 15 new cases per 100,000 population per year, China, Japan and Korea now have up to 80 new cases per 100,000 population per year [2].

Vitamin D is a secosteroid hormone critical to skeletal health and other biological pathways [3]. Vitamin D_3 _is the natural form of vitamin D produced in skin through ultraviolet irradiation of 7-dehydrocholesterol. It is biologically inert and must be metabolized to 25-hydroxyvitamin D_3 _in the liver and then to 1,25-dihydroxyvitamin D_3 _(VD3) in the kidney before functioning [4]. Earlier studies showed that 1,25-dihydroxyvitamin D_3_, the physiologically active form of vitamin D, could induce differentiation and cell cycle arrest in a number of malignant cells, including those in myeloid leukemia, and breast, prostate, colon, skin and brain cancer [5]. VD3 can be antiproliferative in cells of the skin, colon, breast, and prostate, among others, and may also limit proinflammatory stresses [6]. Functional vitamin D receptor (VDR) elements have been identified in the promoter of PTEN, suggesting that vitamin D may play a role in the regulation of PTEN expression [7]. Moreover, it had been demonstrated that VD3 significantly promoted apoptosis in the undifferentiated gastric cancer cell line HGC-27, which was accompanied by a concurrent increase in phosphatase and tensin homolog deletion on chromosome 10 (PTEN) expression with VD3 treatment [8].

Serum 25-hydroxyvitamin D level is the best indicator of overall vitamin D status because it reflects total vitamin D from sunlight exposure, dietary intake, and conversion from adipose stores in the liver [9-11].

Vitamin D deficiency has long been recognized as a medical condition characterized by muscle weakness, ostealgia, and fragility fractures. Vitamin D insufficiency without overt clinical symptoms has recently become a concern of physicians and patients [4]. Generally, vitamin D deficiency refers to a serum level of 25-hydroxyvitamin D below 50 nmol/L, and vitamin D insufficiency 50 to 75 nmol/L. A number of studies have been done to prove whether vitamin D has the preventive function to various kinds of cancers. Results were debatable, and consistent associations have only been demonstrated in colorectal cancer [12,13]. The Cohort Consortium Vitamin D Pooling Project of Rarer Cancers have suggested that circulating 25(OH)D concentration was not significantly associated with upper GI cancer risk, but analysis on race subgroup in that study showed that among Asians, lower concentrations of 25(OH)D were associated with a statistically significant decreased risk of upper GI cancer [14]. A prospective study built an index from factors that predicted higher vitamin D status were statistically significantly associated with a lower risk of esophageal cancer and non-statistically-significantly with a lower risk of stomach cancer [15]. Another study found that higher serum 25(OH)D concentrations were associated with increased risk of esophageal squamous cell carcinoma (ESCC) in men, but not gastric cardia or noncardia adenocarcinoma [16]. Case-control studies of upper GI cancer examining dietary and/or supplemental vitamin D have reported that higher vitamin D intake is associated with lower risk of ESCC [17], increased risk of gastric cancer [18], or had no association with gastric cancer [19]. However, three studies which used different methods----more available solar radiation in lower latitudes [20], higher vitamin D intake [17], and higher vitamin D exposure index [21]----to estimate vitamin D exposure unanimously showed higher vitamin D levels were associated with lower risk of esophageal or stomach cancer.

Though the relationship between Vitamin D status and risk of gastric cancer was indeterminate and a possible relationship has been suggested in many investigations, it has not been identified whether there is a definitive correlation between vitamin D status and clinicopathologic features of gastric cancer patients. Meanwhile, whether Vitamin D status can predict prognosis of patients needs further analyis.

Therefore, a retrospective research of the relationship between serum 25-hydroxyvitamin D and clinicopathologic characteristics of patients with gastric cancer was performed. Besides, we investigated the prognostic significance of serum 25-hydroxyvitamin D in series of gastric cancer patients.

## Methods

### Study population

197 gastric carcinoma patients who had been diagnosed by pathology and received treatment in the cancer center of Sun Yat-sen University from January 2002 to January 2006 were retrospectively analyzed in this study. Blood from them was collected after diagnosis and before any kind of treatment. Subjects who had prior cancer history, daily vitamin D supplementation or diseases which would affect serum 25-hydroxyvitamin D were excluded from the study, including hyperthyroidism, malabsorption, rickets, osteomalacia, hypercortisolism, serious liver disease (defined as liver function Child-pugh classification B or C), renal failure (defined as serum creatinine more than 177 umol/L), and alcoholism. Sera were separated from the venous blood samples by centrifugation, then aliquoted and stored at -80°C until recent use. Clinicopathologic variables and overall survival dates were gained from medical records. Cancer stage was classified according to the 7th editions of the UICC TNM staging systems. Written informed consent from all subjects and approval from the independent Institute Research Ethics Committee at Cancer Center of Sun Yat-sen University were obtained.

### Laboratory analysis

Serum 25-hydroxyvitamin D concentrations were obtained with 25-hydroxy Vitamin D elisa assay (UK Immunodiagnostic Systems Linited). For assessment of assay reliability, each plate had double calibrators to produce standard curve and two control materials. A total of 197 specimens were measured. The identities of all sample sources were blind to the laboratory personnel. Coefficients of variation for the calibrators, high control and low control were 2.77%, 6.28% and 12.97%, respectively.

### Statistical analyses

All the statistical analyses were carried out with the SPSS16.0 statisitical software. P values were derived from 2-sided tests, and those less than 0.05 were considered statistically significant. One way analysis of variance was used to analyze the relationship between the serum 25-hydroxyvitamin D level and clinicopathological characteristics. Kaplan-Meier method was used to plot survival curve with log-rank testing for univariate analysis which included gender, age, T stage, N stage, M stage, tumor size, tumor location, Borrmann type, tumor diffirentiation, chemotherapy, symptom duration(from the time when patient felt discomfortable to diagnosis), season, BMI(body mass index), smoking and drinking. The univariables tended to be associated with survival (*P *< 0.05) were chosen to be analyzed by the Cox proportional hazards model in the multivariate analysis.

## Results

The mean vitamin D level of 197 gastric cancer patients was 49.85 ± 23.68 nmol/L, ranging from 7.26 to 260.47 nmol/L. None of the results were in the intoxicated range (≥374 nmol/L) [22]. According to other researches [23-25], vitamin D levels were divided into three groups: <50 nmol/L as deficient , 50-75 nmol/L as insufficient, >75 nmol/L as sufficient. Thus the mean level of subjects in the National Health and Nutrition Examination Survey fell into insufficient group [26]. The results of this study were as follows: deficient in 114 patients (57.9%), insufficient in 67 patients (34%), and sufficient in 16 patients (8.1%).

The relationship between the serum 25-hydroxyvitamin D level and clinicopathological characteristics was showed in Table 1. There were 134 males and 63 females in this study, and the mean Vitamin D levels were 50.73 ± 18.72 nmol/L in males while 47.96 ± 31.88 nmol/L in females, which showed no significant difference. It suggested an insignificant tendency (*P *= 0.073) that the patients older than 60 years old had higher vitamin D levels. Vitamin D levels had a significant relationship with the season of blood draw (*P *= 0.002), when summer it had the highest levels and winter the lowest levels. Clinical stage (*P *= 0.004) and lymph node metastasis classification (*P *= 0.009) were significantly associated with vitamin D levels. Meanwhile, T classification (*P *= 0.071) and distant metastasis (*P *= 0.062) tended to be insignificantly associated with the vitamin D levels. However, there was no significant correlation between the vitamin D levels and tumor size, tumor position, pathologic differentiation, Borrmann type, symptom duration, BMI, smoking or drinking.

**Table 1:** Correlation between patient's clinicopathologic characteristics and vitamin D levels

Variable	No.(%)	Vitamin D(nmol/l) mean±SD	P-value
Gender			0.445
Male	134 (68.0)	50.73 ± 18.72	
Female	63 (32.0)	47.96 ± 31.88	

Age(years)			0.073
<60	101 (51.3)	46.90 ± 18.24	
≥60	96 (48.7)	52.94 ± 28.07	

Tumor size(cm)			0.143
<5.0	102 (53.4)	48.12 ± 16.21	
≥5.0	89 (46.6)	53.13 ± 29.74	

Tumor position			0.462
Cardia/gastric fundus	59 (29.9)	49.36 ± 16.11	
Gastric body	50 (25.4)	46.76 ± 17.52	
Gastric antrum/pylorus	88 (44.7)	51.93 ± 30.13	

Differentiation			0.957
Well/moderate	30 (15.2)	49.63 ± 13.43	
Poor	167 (84.8)	49.89 ± 25.11	

Borrmann type			0.847
I/II	73 (37.1)	50.27 ± 16.04	
III/IV	124 (62.9)	49.60 ± 27.25	

Clinical stage			0.004
I/II	75 (38.1)	56.05 ± 29.94	
III/IV	122 (61.9)	46.03 ± 17.93	

T classification			0.071
T1	17 (8.7)	47.53 ± 10.01	
T2	23 (11.8)	56.25 ± 20.07	
T3	112 (57.4)	52.07 ± 27.67	
T4	43 (22.1)	42.43 ± 15.29	

N classification			0.009
N0/N1	76 (40.0)	55.98 ± 29.26	
N2/N3	114 (60.0)	46.81 ± 18.63	

Distant metastasis			0.062
Positive	29 (14.7)	51.15 ± 24.32	
Negative	168 (85.3)	42.28 ± 18.16	

Symptom duration(month)			0.494
<4	96 (49.2)	48.82 ± 19.62	
≥4	99 (50.8)	51.16 ± 27.19	

Season of blood draw			0.002
Spring(Apr-Jun)	36 (18.3)	49.06 ± 34.49	
Summer(Jul-Sep)	52 (26.4)	58.68 ± 19.78	
Autumn(Oct-Dec)	53 (26.9)	48.86 ± 15.55	
Winter(Jan-Mar)	56 (28.4)	39.52 ± 14.68	

BMI(kg/m2)			0.458
<25.0	158 (81.0)	50.61 ± 25.12	
≥25.0	37 (19.0)	47.39 ± 16.58	

Smoking daily			0.818
Yes	73 (37.4)	50.51 ± 20.78	
No	122 (62.6)	49.70 ± 25.42	

Drinking daily			0.328
Yes	24 (12.3)	45.56 ± 14.18	
No	171 (87.7)	50.01 ± 23.74	

**Figure 1:**
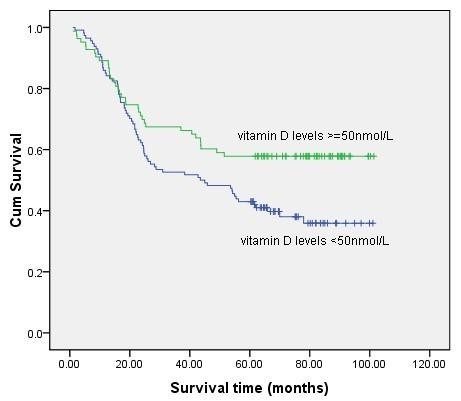
**Comparison of survival rates in patients with high and low vitamin D levels using Kaplan-Meier curves with univariate analysis **.

Among the 197 gastric cancer patients, 106 (53.8%) died during follow-up of more than 5 years, all of whom died of cancer recurrence or progress. Kaplan-Meier survival analysis with log-rank statistics was used to determine the association between the vitamin D levels and overall survival. The univariate analysis showed that the high vitamin D levels group (≥ 50 nmol/L) was associated with improved 5-year overall survival compared with the low vitamin D levels group (< 50 nmol/L). According to the log-rank test (*P *= 0.018), there was a significant difference in overall survival between these two groups (Figure 1). The 5-year survival rate was 57.8% in high vitamin D levels group and 43% in the low vitamin D levels group. The mean survival time was 67.3 months and 54.0 months in high and low vitamin D group respectively.

Kaplan-Meier analysis and the log-rank test were also used to evaluate the effect of clinicopathological characteristics (Table 2), including gender, age, T classification, N classification, distant metastasis, tumor size, tumor position, Borrmann type, tumor diffirentiation, chemical therapy, duration from the start of symptom to diagnosis, season, BMI(body mass index), smoking and drinking. Results of univariate analysis showed tumor size (*P *< 0.001), tumor position (*P *= 0.011), Borrmann type (*P *= 0.001), T classification (*P *< 0.001), N classification (*P *< 0.001), distant metastasis (*P *< 0.001) and symptom duration (*P *= 0.001) were significantly correlated with survival. The other factors did not have significant relationship with the survival. The Cox proportional hazards mode was used to test the independent effects of all the above significant factors. In this exploratory multivariate analyses, vitamin D levels (*P *= 0.019), tumor size (*P *= 0.026), tumor position (*P *< 0.001), T classification (*P *= 0.001), N classification (*P *= 0.003) and distant metastasis (*P *< 0.001) were recognized as independent prognostic factors, while Borrmann type (*P *= 0.135) and symptom duration (*P *= 0.067) were not independent predictors. Therefore, our findings indicate that vitamin D level has a significant correlation with the prognosis of gastric cancer.

**Table 2:** Univariate and multivariate analysis of different prognostic Variables for overall survival in gastric cancer patients by Cox regression analysis

	Univariate analysis		Multivariate analysis		
	No. of patients	P-value	Regression coefficient(SE)	Hazard ratios(95% confidence internal)	*P*-value

Vitamin D status		0.018	-0.536(0.228)	0.585(0.374-0.914)	0.019

< 50 nmol/L	114				

≥ 50 nmol/L	83				

Tumor size		< 0.001	0.523(0.235)	1.687(1.064-2.673)	0.026

< 5 cm	102				

≥ 5 cm	89				

Tumor position		0.011	-0.540(0.148)	0.583(0.436-0.779)	<0.001

Cardia/gastric fundus	59				

Gastric body	50				

Gastric antrum/pylorus	88				

Borrmann type		0.001	-0.392(0.262)	0.676(0.404-1.129)	0.135

I/II	73				

III/IV	124				

T classification		<0.001	1.891(0.567)	6.623(2.181-20.115)	0.001

T1/T2	41				

T3/T4	154				

N classification		<0.001	0.859(0.292)	2.362(1.333-4.186)	0.003

N0/N1	76				

N2/N3	114				

Distant metastasis		<0.001	2.374(0.352)	10.739(5.383-21.424)	<0.001

Positive	29				

Negative	168				

Symptom duration		0.001	-0.435(0.238)	0.647(0.406-1.031)	0.067

< 4 months	96				

≥ 4 months	99				

## Discussion

In our study, we first assayed the vitamin D status in gastric cancer patients, and found out that only 8.1% patients reached the sufficient level while up to 57.9% patients were in the deficient level. It has been reported that vitamin D levels of breast cancer patients were sufficient in 24% patients and deficient in 37.5% [27], whose overall results were better than that of gastric cancer. 25-hydroxyvitamin D has been detected in the healthy Asians and the mean was 59.50 ± 25.25 nmol/L [28], which was higher than the result 49.85 ± 23.68 nmol/L in our study.

Then we combined the vitamin D level with clinicopathological data, which showed that serum 25-hydroxyvitamin D concentrations have been inversely associated with clinical stage and lymph node metastasis. This finding is similar to the results of some clinical studies, in which serum 25-hydroxyvitamin D concentrations have been inversely associated with breast cancer stage, for instance [29,30]. It was found that circulating 25-hydroxyvitamin D concentrations were inversely associated with BMI, as patients with BMI higher than 30 kg/m^2 ^had lower vitamin D levels [23,27]. However, we did not arrive at this result because in our study patients' BMI were seldom higher than 30 kg/m^2^, so we adjusted the cutting point at 25 kg/m^2^. It could be expected that vitamin D levels of the blood samples drawn in summer was the highest, as summer offers adequate sunshine, the major determinant of vitamin D status in humans.

Finally, low vitamin D levels were significantly associated with poor survival in univariate analysis. The vitamin D levels were demonstrated to be an independent prognostic factor of gastric cancer by the Cox proportional hazards model in the multivariate analysis. In patients with breast cancer [27], colorectal cancer [31] or early-stage non-small-cell lung cancer [32], other studies have also reached the same conclusion that higher 25 OHD levels were associated with better prognosis. We also found that tumor position was an important prognostic factor, as was explained by the extent of operation that was required. Patients who underwent subtotal gastrectomy had a significantly longer median overall survival than those who underwent a total gastrectomy [33].

Recent findings from Intergroup Trial N9741 revealed that stage IV colorectal cancer patients randomly assigned to FOLFOX have improved survival with higher baseline levels of 25-hydroxyvitamin D [25]. Lower cancer survival rates among black patients may be due to lower serum 25-hydroxyvitamin D, whose production from UVB irradiance is lower because of darker skin [34]. Vitamin D production in the skin seems to decrease the risk of several solid cancers (especially stomach, colorectal, liver and gallbladder, pancreas, lung, female breast, prostate, bladder and kidney cancers) [35].

Though the explicit biological mechanisms are not clear, some data provides a potential explanation for our findings. VD3 could induce differentiation and cell cycle arrest in a number of malignant cells, including those in myeloid leukemia, and breast, prostate, colon, skin and brain cancer [4,5,36,37]. It has been reported that VD3 (the active form of vitamin D) significantly promoted apoptosis in the undifferentiated gastric cancer cell line HGC-27 [8]. Vitamin D may prevent gastric cancers from progressing by modulating the extracellular microenvironment, as vitamin D has been shown to alter the expression of multiple genes in the extracelluar matrix remodeling [38,39]. VD3 can inhibit Wnt signaling by interrupting the crosstalk between tumor epithelial cells and its microenvironment [40]. Functional vitamin D receptor (VDR) elements have been identified in the promoter of PTEN, suggesting that vitamin D may play a role in the regulation of PTEN expression [7]. In undifferentiated colon cancer cells CYP24A1 (a key enzyme in vitamin D metabolism) expression was increased compared with normal cells [41], so it potentially prevented the synthesis of 1,25-dihydroxyvitamin D_3 _and restricted its cancer protective effects. What's more, vitamin D could also regulate the phenotype of human breast cancer cells [42].

It was suggested that gastrectomy moderately influenced the metabolism of vitamin D, and vitamin D level in patients was significantly lower at 1 year or more postoperatively than at less than 1 year postoperatively, especially in those who had received total gastrectomy [43]. From the perspective of a gastroenterologist, Vitamin D supplementation is recommended for persons with disorders of malabsorption and cholestasis [44]. High doses (1,100 IU) of vitamin D plus calcium were shown to significantly reduce cancer incidence in women [45]. From all the evidence above, perhaps it is reasonable for vitamin D deficient patients to have appropriate supplementation, however, clinical trials which use high doses of vitamin D are needed to determine whether vitamin D really improves survival.

One limitation of our study is that the vitamin D levels were measured only once at diagnosis, which may not totally represent the vitamin D levels during cancer generation or progression. In another study measuring the serum 25-hydroxyvitamin D concentrations, in breast cancer patients its correlation coefficient in 1994 and 2008 ranged from 0.42 to 0.52, when measured 12 months respectively it was 0.80 [42], which suggested the stability of endogenous vitamin D status. The blood samples of our study were collected once after diagnosis, before any medical therapy or surgery, so routine life behavior change was unlikely to affect vitamin D status.

## Conclusions

In conclusion, our study provides evidence that serum vitamin D level is a significant independent prognostic factor in gastric cancer patients, and vitamin D deficiency may be associated with poor prognosis. We also describe the correlation between vitamin D level and clinicopathological characteristics. All the findings require larger scale prospective studies to confirm. In addition, the mechanism of potential beneficial effects of vitamin D in gastric cancer needs further study. Considering all the existing evidence and the fact that deficient vitamin D status can be easily corrected by taking supplement or increasing sunlight exposure as well, we cautiously suggest clinicians to evaluate the vitamin D status of gastric cancer patients, and then consider appropriate vitamin D supplementation to deficient patients.

## Competing interests

We have no financial or personal relationships with other people or organizations that would bias our work. No benefits in any form have been received or will be received from a commercial party related directly or indirectly to the subject of our article.

## Authors' contributions

RC conceived the study, carried out the Elisa, participated in collecting clinical data of the gastric cancer patients and drafted the manuscript. QMZ carried out the Elisa, WDS collected clinical data. LHY performed statistical analysis. ZDS and WZQ participated in the design of the study. WFH and LYH drafted the manuscript and participated in the statistical analysis. ZZW participated in collecting blood samples. XRH participated in its design and coordination and helped to draft the manuscript.
